# Supplementary material for: Overlapping cell population expression profiling and regulatory inference in C. elegans
Source: BMC Genomics. 2016 Feb 29;17:159. doi: 10.1186/s12864-016-2482-z (PMC4772325; doi:10.1186/s12864-016-2482-z)
Supplement: Additional file 13: — Web supplement. (DOC 21 kb) [file 12864_2016_2482_MOESM13_ESM.zip › sortWeb/clusters/hier.300.clusters/6.html]

Cluster 6 

## Cluster 6

### Expression

| cnd-1 rep. 1 | cnd-1 rep. 2 | cnd-1 rep. 3 | pha-4 rep. 1 | pha-4 rep. 2 | pha-4 rep. 3 | ceh-27 | ceh-36 | ceh-6 | F21D5.9 | mir-57 | mls-2 | pal-1 | pros-1 | ttx-3 | unc-130 | hlh-16 | irx-1 | ceh-6 (+) hlh-16 (+) | ceh-6 (+) hlh-16 (-) | ceh-6 (-) hlh-16 (+) | cnd-1 singlets | pha-4 singlets | 0 | 60 | 120 | 150 | 180 | 240 | 330 | 390 | 420 | 480 | 540 | 570 | 600 | 630 | 660 | NAME | Functional description |
| --- | --- | --- | --- | --- | --- | --- | --- | --- | --- | --- | --- | --- | --- | --- | --- | --- | --- | --- | --- | --- | --- | --- | --- | --- | --- | --- | --- | --- | --- | --- | --- | --- | --- | --- | --- | --- | --- | --- | --- |
|  |  |  |  |  |  |  |  |  |  |  |  |  |  |  |  |  |  |  |  |  |  |  |  |  |  |  |  |  |  |  |  |  |  |  |  |  |  | F09G2.2 |  |
|  |  |  |  |  |  |  |  |  |  |  |  |  |  |  |  |  |  |  |  |  |  |  |  |  |  |  |  |  |  |  |  |  |  |  |  |  |  | *taf-13* | TAF (TBP-associated transcription factor) family |
|  |  |  |  |  |  |  |  |  |  |  |  |  |  |  |  |  |  |  |  |  |  |  |  |  |  |  |  |  |  |  |  |  |  |  |  |  |  | F41C3.4 |  |
|  |  |  |  |  |  |  |  |  |  |  |  |  |  |  |  |  |  |  |  |  |  |  |  |  |  |  |  |  |  |  |  |  |  |  |  |  |  | C28H8.4 |  |
|  |  |  |  |  |  |  |  |  |  |  |  |  |  |  |  |  |  |  |  |  |  |  |  |  |  |  |  |  |  |  |  |  |  |  |  |  |  | *trap-4* | TRanslocon-Associated Protein |
|  |  |  |  |  |  |  |  |  |  |  |  |  |  |  |  |  |  |  |  |  |  |  |  |  |  |  |  |  |  |  |  |  |  |  |  |  |  | Y75B8A.6 |  |
|  |  |  |  |  |  |  |  |  |  |  |  |  |  |  |  |  |  |  |  |  |  |  |  |  |  |  |  |  |  |  |  |  |  |  |  |  |  | B0250.5 |  |
|  |  |  |  |  |  |  |  |  |  |  |  |  |  |  |  |  |  |  |  |  |  |  |  |  |  |  |  |  |  |  |  |  |  |  |  |  |  | T21H8.5 |  |
|  |  |  |  |  |  |  |  |  |  |  |  |  |  |  |  |  |  |  |  |  |  |  |  |  |  |  |  |  |  |  |  |  |  |  |  |  |  | *trap-1* | TRanslocon-Associated Protein |
|  |  |  |  |  |  |  |  |  |  |  |  |  |  |  |  |  |  |  |  |  |  |  |  |  |  |  |  |  |  |  |  |  |  |  |  |  |  | *tsn-1* | Tudor Staphylococcal Nuclease homolog |
|  |  |  |  |  |  |  |  |  |  |  |  |  |  |  |  |  |  |  |  |  |  |  |  |  |  |  |  |  |  |  |  |  |  |  |  |  |  | F07H5.13 |  |
|  |  |  |  |  |  |  |  |  |  |  |  |  |  |  |  |  |  |  |  |  |  |  |  |  |  |  |  |  |  |  |  |  |  |  |  |  |  | *mpst-3* | MercaptoPyruvate SulfurTransferase homolog |
|  |  |  |  |  |  |  |  |  |  |  |  |  |  |  |  |  |  |  |  |  |  |  |  |  |  |  |  |  |  |  |  |  |  |  |  |  |  | K08H2.4 |  |
|  |  |  |  |  |  |  |  |  |  |  |  |  |  |  |  |  |  |  |  |  |  |  |  |  |  |  |  |  |  |  |  |  |  |  |  |  |  | *pdi-1* | Protein Disulfide Isomerase |
|  |  |  |  |  |  |  |  |  |  |  |  |  |  |  |  |  |  |  |  |  |  |  |  |  |  |  |  |  |  |  |  |  |  |  |  |  |  | *rhi-1* | RHo GDI |
|  |  |  |  |  |  |  |  |  |  |  |  |  |  |  |  |  |  |  |  |  |  |  |  |  |  |  |  |  |  |  |  |  |  |  |  |  |  | F52A8.5 |  |
|  |  |  |  |  |  |  |  |  |  |  |  |  |  |  |  |  |  |  |  |  |  |  |  |  |  |  |  |  |  |  |  |  |  |  |  |  |  | ZK632.12 |  |
|  |  |  |  |  |  |  |  |  |  |  |  |  |  |  |  |  |  |  |  |  |  |  |  |  |  |  |  |  |  |  |  |  |  |  |  |  |  | *nuo-6* | NADH Ubiquinone Oxidoreductase |
|  |  |  |  |  |  |  |  |  |  |  |  |  |  |  |  |  |  |  |  |  |  |  |  |  |  |  |  |  |  |  |  |  |  |  |  |  |  | F17C11.4 |  |
|  |  |  |  |  |  |  |  |  |  |  |  |  |  |  |  |  |  |  |  |  |  |  |  |  |  |  |  |  |  |  |  |  |  |  |  |  |  | *msp-78* | Major Sperm Protein |
|  |  |  |  |  |  |  |  |  |  |  |  |  |  |  |  |  |  |  |  |  |  |  |  |  |  |  |  |  |  |  |  |  |  |  |  |  |  | T19B4.3 |  |
|  |  |  |  |  |  |  |  |  |  |  |  |  |  |  |  |  |  |  |  |  |  |  |  |  |  |  |  |  |  |  |  |  |  |  |  |  |  | K04G2.7 |  |
|  |  |  |  |  |  |  |  |  |  |  |  |  |  |  |  |  |  |  |  |  |  |  |  |  |  |  |  |  |  |  |  |  |  |  |  |  |  | T19D7.6 |  |
|  |  |  |  |  |  |  |  |  |  |  |  |  |  |  |  |  |  |  |  |  |  |  |  |  |  |  |  |  |  |  |  |  |  |  |  |  |  | C17E4.11 |  |
|  |  |  |  |  |  |  |  |  |  |  |  |  |  |  |  |  |  |  |  |  |  |  |  |  |  |  |  |  |  |  |  |  |  |  |  |  |  | H43I07.3 |  |
|  |  |  |  |  |  |  |  |  |  |  |  |  |  |  |  |  |  |  |  |  |  |  |  |  |  |  |  |  |  |  |  |  |  |  |  |  |  | *spg-7* | human SPG (spastic paraplegia) |
|  |  |  |  |  |  |  |  |  |  |  |  |  |  |  |  |  |  |  |  |  |  |  |  |  |  |  |  |  |  |  |  |  |  |  |  |  |  | T28D6.6 |  |
|  |  |  |  |  |  |  |  |  |  |  |  |  |  |  |  |  |  |  |  |  |  |  |  |  |  |  |  |  |  |  |  |  |  |  |  |  |  | *ostb-1* | OligoSaccharylTransferase, Beta subunit |
|  |  |  |  |  |  |  |  |  |  |  |  |  |  |  |  |  |  |  |  |  |  |  |  |  |  |  |  |  |  |  |  |  |  |  |  |  |  | T24A6.20 |  |
|  |  |  |  |  |  |  |  |  |  |  |  |  |  |  |  |  |  |  |  |  |  |  |  |  |  |  |  |  |  |  |  |  |  |  |  |  |  | Y53C12A.3 |  |
|  |  |  |  |  |  |  |  |  |  |  |  |  |  |  |  |  |  |  |  |  |  |  |  |  |  |  |  |  |  |  |  |  |  |  |  |  |  | *fbxa-76* | F-box A protein |
|  |  |  |  |  |  |  |  |  |  |  |  |  |  |  |  |  |  |  |  |  |  |  |  |  |  |  |  |  |  |  |  |  |  |  |  |  |  | F18E9.10 |  |
|  |  |  |  |  |  |  |  |  |  |  |  |  |  |  |  |  |  |  |  |  |  |  |  |  |  |  |  |  |  |  |  |  |  |  |  |  |  | F16B12.5 |  |
|  |  |  |  |  |  |  |  |  |  |  |  |  |  |  |  |  |  |  |  |  |  |  |  |  |  |  |  |  |  |  |  |  |  |  |  |  |  | R02F2.9 |  |
|  |  |  |  |  |  |  |  |  |  |  |  |  |  |  |  |  |  |  |  |  |  |  |  |  |  |  |  |  |  |  |  |  |  |  |  |  |  | D2024.17 |  |
|  |  |  |  |  |  |  |  |  |  |  |  |  |  |  |  |  |  |  |  |  |  |  |  |  |  |  |  |  |  |  |  |  |  |  |  |  |  | ZK112.4 |  |
|  |  |  |  |  |  |  |  |  |  |  |  |  |  |  |  |  |  |  |  |  |  |  |  |  |  |  |  |  |  |  |  |  |  |  |  |  |  | C16E9.11 |  |
|  |  |  |  |  |  |  |  |  |  |  |  |  |  |  |  |  |  |  |  |  |  |  |  |  |  |  |  |  |  |  |  |  |  |  |  |  |  | F57B7.8 |  |
|  |  |  |  |  |  |  |  |  |  |  |  |  |  |  |  |  |  |  |  |  |  |  |  |  |  |  |  |  |  |  |  |  |  |  |  |  |  | T07F10.11 |  |
|  |  |  |  |  |  |  |  |  |  |  |  |  |  |  |  |  |  |  |  |  |  |  |  |  |  |  |  |  |  |  |  |  |  |  |  |  |  | C15B12.11 |  |
|  |  |  |  |  |  |  |  |  |  |  |  |  |  |  |  |  |  |  |  |  |  |  |  |  |  |  |  |  |  |  |  |  |  |  |  |  |  | F42H10.11 |  |
|  |  |  |  |  |  |  |  |  |  |  |  |  |  |  |  |  |  |  |  |  |  |  |  |  |  |  |  |  |  |  |  |  |  |  |  |  |  | C01B7.3 |  |
|  |  |  |  |  |  |  |  |  |  |  |  |  |  |  |  |  |  |  |  |  |  |  |  |  |  |  |  |  |  |  |  |  |  |  |  |  |  | D1053.5 |  |
|  |  |  |  |  |  |  |  |  |  |  |  |  |  |  |  |  |  |  |  |  |  |  |  |  |  |  |  |  |  |  |  |  |  |  |  |  |  | R12E2.11 |  |
|  |  |  |  |  |  |  |  |  |  |  |  |  |  |  |  |  |  |  |  |  |  |  |  |  |  |  |  |  |  |  |  |  |  |  |  |  |  | *prdx-6* | PeRoxireDoXin |
|  |  |  |  |  |  |  |  |  |  |  |  |  |  |  |  |  |  |  |  |  |  |  |  |  |  |  |  |  |  |  |  |  |  |  |  |  |  | *dod-18* | Downstream Of DAF-16 (regulated by DAF-16) |
|  |  |  |  |  |  |  |  |  |  |  |  |  |  |  |  |  |  |  |  |  |  |  |  |  |  |  |  |  |  |  |  |  |  |  |  |  |  | *mrpl-9* | Mitochondrial Ribosomal Protein, Large |
|  |  |  |  |  |  |  |  |  |  |  |  |  |  |  |  |  |  |  |  |  |  |  |  |  |  |  |  |  |  |  |  |  |  |  |  |  |  | R151.10 |  |
|  |  |  |  |  |  |  |  |  |  |  |  |  |  |  |  |  |  |  |  |  |  |  |  |  |  |  |  |  |  |  |  |  |  |  |  |  |  | *mrps-6* | Mitochondrial Ribosomal Protein, Small |
|  |  |  |  |  |  |  |  |  |  |  |  |  |  |  |  |  |  |  |  |  |  |  |  |  |  |  |  |  |  |  |  |  |  |  |  |  |  | *mrps-18B* | Mitochondrial Ribosomal Protein, Small |
|  |  |  |  |  |  |  |  |  |  |  |  |  |  |  |  |  |  |  |  |  |  |  |  |  |  |  |  |  |  |  |  |  |  |  |  |  |  | Y116A8C.27 |  |
|  |  |  |  |  |  |  |  |  |  |  |  |  |  |  |  |  |  |  |  |  |  |  |  |  |  |  |  |  |  |  |  |  |  |  |  |  |  | *mrpl-21* | Mitochondrial Ribosomal Protein, Large |
|  |  |  |  |  |  |  |  |  |  |  |  |  |  |  |  |  |  |  |  |  |  |  |  |  |  |  |  |  |  |  |  |  |  |  |  |  |  | *tsfm-1* | TS elongation Factor (EF-Ts), Mitochondrial |
|  |  |  |  |  |  |  |  |  |  |  |  |  |  |  |  |  |  |  |  |  |  |  |  |  |  |  |  |  |  |  |  |  |  |  |  |  |  | *mrpl-49* | Mitochondrial Ribosomal Protein, Large |
|  |  |  |  |  |  |  |  |  |  |  |  |  |  |  |  |  |  |  |  |  |  |  |  |  |  |  |  |  |  |  |  |  |  |  |  |  |  | C34C12.8 |  |
|  |  |  |  |  |  |  |  |  |  |  |  |  |  |  |  |  |  |  |  |  |  |  |  |  |  |  |  |  |  |  |  |  |  |  |  |  |  | *mrpl-54* | Mitochondrial Ribosomal Protein, Large |
|  |  |  |  |  |  |  |  |  |  |  |  |  |  |  |  |  |  |  |  |  |  |  |  |  |  |  |  |  |  |  |  |  |  |  |  |  |  | T09A5.5 |  |
|  |  |  |  |  |  |  |  |  |  |  |  |  |  |  |  |  |  |  |  |  |  |  |  |  |  |  |  |  |  |  |  |  |  |  |  |  |  | F45H11.8 |  |
|  |  |  |  |  |  |  |  |  |  |  |  |  |  |  |  |  |  |  |  |  |  |  |  |  |  |  |  |  |  |  |  |  |  |  |  |  |  | *mrpl-23* | Mitochondrial Ribosomal Protein, Large |
|  |  |  |  |  |  |  |  |  |  |  |  |  |  |  |  |  |  |  |  |  |  |  |  |  |  |  |  |  |  |  |  |  |  |  |  |  |  | C06G3.8 |  |
|  |  |  |  |  |  |  |  |  |  |  |  |  |  |  |  |  |  |  |  |  |  |  |  |  |  |  |  |  |  |  |  |  |  |  |  |  |  | *exos-4.2* | EXOSome (multiexonuclease complex) component |
|  |  |  |  |  |  |  |  |  |  |  |  |  |  |  |  |  |  |  |  |  |  |  |  |  |  |  |  |  |  |  |  |  |  |  |  |  |  | F10E7.6 |  |
|  |  |  |  |  |  |  |  |  |  |  |  |  |  |  |  |  |  |  |  |  |  |  |  |  |  |  |  |  |  |  |  |  |  |  |  |  |  | F08F8.7 |  |

### Phenotypes enriched

none found

### Anatomy terms enriched

none found

### GO terms enriched

|  |  |  |
| --- | --- | --- |
| **GO term** | **Number of genes** | **FDR-corrected p-value** |
| cytoplasm | 18 | 1.8e-05 |
| vesicle-mediated transport | 11 | 1.2e-02 |
| mitochondrion | 6 | 2.3e-02 |
| receptor-mediated endocytosis | 9 | 3.1e-02 |
| structural constituent of ribosome | 4 | 4.8e-02 |

### Expression clusters enriched

|  |  |  |  |
| --- | --- | --- | --- |
| **Group name** | **Number in cluster** | **Enrichment** | **FDR corrected p** |
| Genes in the top 10% of expression level across the triplicate L3 samples. To generate the top10 and bottom10 gene sets, authors ranked all genes by mean expression array signal intensity across the three replicates, then took the top and bottom deciles (1,841 genes each) to represent genes with high and low expression. | 20 | 2.71 | 0.00579 |
| Genes with no change in hcf-1(-), no change in sir-2.1(O/E) and downregulated in daf-2(-). | 12 | 4.13 | 0.00731 |
| Caenorhabditis elegans Genes with expression levels changed significantly after treatment of Bacillus thurigiensis DB27. | 34 | 1.77 | 0.02150 |

### Motifs enriched

|  |  |  |  |  |  |
| --- | --- | --- | --- | --- | --- |
| **Motif** | **Logo** | **Possible orthologs** | **Number of motifs in cluster** | **Enrichment** | **FDR corrected p** |
| V$XFD1\_01 |  | let-381 lin-31 | 48 | 1.57 | 0.0011 |
| pTH10030 |  | xbp-1 (-0.58) | 19 | 3.01 | 0.0014 |
| V$XFD3\_01 |  | let-381 ceh-20 | 48 | 1.56 | 0.0016 |
| pTH9222 |  | mel-28 | 48 | 1.55 | 0.0017 |
| EHF\_si |  | C24A1.2 | 41 | 1.70 | 0.0024 |
| Dll\_Cell\_FBgn0000157 |  | ceh-43 | 41 | 1.66 | 0.0042 |
| ONECUT3\_1 |  | ceh-48 | 53 | 1.40 | 0.0046 |
| V$TBP\_01 |  | tbp-1 | 44 | 1.57 | 0.0053 |
| HLH30 |  | hlh-30 | 27 | 2.08 | 0.0065 |
| Fkh2 |  | fkh-10 (-0.55) | 46 | 1.50 | 0.0080 |
| V$RORA1\_01 |  | nhr-213 | 35 | 1.76 | 0.0080 |
| PHOX2B\_1 |  | alr-1 | 42 | 1.58 | 0.0086 |
| pTH10624 |  | Y61A9LA.9 Y116A8C.22 | 44 | 1.52 | 0.0120 |
| POU3F4\_1 |  | ceh-18 | 24 | 2.12 | 0.0130 |
| pTH9226 |  | daf-19 (-0.62) | 15 | 2.88 | 0.0140 |
| pTH9116 |  | fkh-7 (0.58) daf-16 let-381 lin-31 | 46 | 1.46 | 0.0140 |
| MA0508.1 |  | blmp-1 | 50 | 1.39 | 0.0150 |
| MA0457.1 |  | alr-1 lim-6 | 41 | 1.56 | 0.0150 |
| pTH9082 |  | mab-23 | 49 | 1.41 | 0.0160 |
| pTH5516 |  | nhr-2 nhr-213 | 25 | 2.03 | 0.0170 |
| pTH10026 |  | ceh-32 ceh-20 F55C5.11 | 30 | 1.83 | 0.0170 |
| GATA3\_2 |  | elt-1 | 15 | 2.82 | 0.0170 |
| MA0244.1 |  | C48E7.11 | 45 | 1.47 | 0.0170 |
| V$TAXCREB\_01 |  | crh-1 | 27 | 1.93 | 0.0180 |
| cad\_FlyReg\_FBgn0000251 |  | ceh-24 ceh-13 | 43 | 1.50 | 0.0180 |
| sqz\_SANGER\_5\_FBgn0010768 |  | fkh-7 (0.58) lin-29 (0.53) | 47 | 1.43 | 0.0190 |
| pTH9097 |  | Y116A8C.22 | 54 | 1.31 | 0.0200 |
| slp2\_SANGER\_5\_FBgn0004567 |  | fkh-10 (-0.55) daf-16 lin-31 | 22 | 2.14 | 0.0210 |
| rn\_SOLEXA\_5\_FBgn0259172 |  | lin-29 (0.53) | 51 | 1.36 | 0.0220 |
| pTH9958 |  | ztf-6 | 44 | 1.47 | 0.0230 |
| V$AREB6\_02 |  | ztf-6 | 11 | 3.46 | 0.0230 |
| ETV3\_1 |  | lin-1 | 21 | 2.17 | 0.0250 |
| SRF\_2 |  | unc-120 | 7 | 5.38 | 0.0250 |
| MA0070.1 |  | ceh-20 | 36 | 1.62 | 0.0260 |
| HAND1\_si |  | hlh-8 | 32 | 1.71 | 0.0270 |
| V$GATA6\_01 |  | elt-1 | 11 | 3.37 | 0.0270 |
| HXD9\_f1 |  | lin-39 | 45 | 1.44 | 0.0270 |
| pTH8985 |  | athp-1 | 37 | 1.58 | 0.0290 |
| pTH10707 |  | elt-1 elt-6 | 20 | 2.20 | 0.0290 |
| pTH9189 |  | dmd-3 | 45 | 1.43 | 0.0290 |
| PTF1A\_f1 |  | hlh-2 (0.57) lin-32 | 30 | 1.75 | 0.0300 |
| Lmx1a\_2238 |  | lim-6 | 33 | 1.66 | 0.0330 |
| pTH10027 |  | M03D4.4 | 25 | 1.91 | 0.0340 |
| pTH9159 |  | atf-6 | 25 | 1.90 | 0.0370 |
| NR2F6\_f1 |  | nhr-2 | 43 | 1.45 | 0.0380 |
| pTH10633 |  | R07H5.10 | 31 | 1.70 | 0.0380 |
| pTH8399 |  | lin-54 | 46 | 1.40 | 0.0400 |
| NR2C1\_si |  | nhr-19 | 30 | 1.71 | 0.0430 |
| HXC6\_f1 |  | lin-39 | 46 | 1.39 | 0.0440 |
| pTH10797 |  | K11D2.4 | 51 | 1.32 | 0.0440 |
| pTH3510 |  | nhr-86 | 25 | 1.86 | 0.0450 |
| V$GATA1\_06 |  | elt-1 | 23 | 1.94 | 0.0460 |
| pTH10721 |  | ztf-9 | 12 | 2.90 | 0.0460 |
| pTH3866 |  | hlh-2 (0.57) | 25 | 1.86 | 0.0470 |
| pTH9879 |  | C27D6.4 | 24 | 1.89 | 0.0500 |

### Correlated (and anti-correlated) transcription factors

|  |  |
| --- | --- |
| **Transcription factor** | **Correlation** |
| duxl-1 | 0.73 |
| ztf-13 | 0.70 |
| nhr-13 | 0.70 |
| pha-2 | 0.68 |
| Y53F4B.3 | 0.68 |
| F27D4.4 | 0.67 |
| nhr-64 | 0.67 |
| ceh-34 | 0.66 |
| sex-1 | 0.65 |
| ces-1 | 0.64 |
| nhr-130 | 0.63 |
| ces-2 | 0.63 |
| nhr-256 | 0.63 |
| ceh-53 | 0.62 |
| pax-1 | 0.62 |
| nhr-42 | 0.62 |
| pop-1 | 0.62 |
| Y82E9BR.17 | 0.62 |
| zip-7 | 0.61 |
| fkh-6 | 0.61 |
| F40G9.14 | 0.61 |
| dnj-11 | 0.59 |
| ceh-60 | 0.59 |
| ceh-16 | 0.58 |
| fkh-7 | 0.58 |
| ctbp-1 | -0.60 |
| Y17G7B.22 | -0.61 |
| ceh-88 | -0.62 |
| nhr-255 | -0.62 |
| sem-4 | -0.62 |
| daf-19 | -0.62 |
| madf-5 | -0.63 |
| ceh-9 | -0.63 |
| ceh-37 | -0.63 |
| unc-42 | -0.63 |
| mgl-2 | -0.64 |
| F10B5.3 | -0.65 |
| nhr-101 | -0.65 |
| lfi-1 | -0.65 |
| mbr-1 | -0.66 |
| ceh-54 | -0.67 |
| ceh-62 | -0.67 |
| ccch-1 | -0.68 |
| dmd-10 | -0.68 |
| gei-3 | -0.69 |
| nhr-26 | -0.70 |
| saeg-1 | -0.71 |
| tag-97 | -0.72 |
| nhr-91 | -0.75 |
| nhr-145 | -0.76 |

### ChIP peaks enriched

|  |  |  |  |  |
| --- | --- | --- | --- | --- |
| **Gene** | **Experiment** | **Number of upstream peaks** | **Enrichment** | **FDR corrected p** |
| dpl-1 | DPL-1\_Fed-L1-stage-larvae | 27 | 2.51 | 8.8e-05 |
| W03F9.2 | W03F9.2\_L4-Young-Adult-stage-larvae | 37 | 1.89 | 2.8e-04 |
| ceh-39 | CEH-39\_Embryos | 21 | 2.84 | 2.9e-04 |
| nhr-25 | NHR-25\_Larvae-L2-stage | 24 | 2.48 | 4.9e-04 |
| nhr-77 | NHR-77\_Fed-L1-stage-larvae | 26 | 2.30 | 6.5e-04 |
| pha-4 | PHA-4\_Larvae-L2-stage | 29 | 2.11 | 8.3e-04 |
| C34F6.9 | C34F6.9\_Larvae-L2-stage | 27 | 2.19 | 1.0e-03 |
| hpl-2 | HPL-2\_Fed-L1-stage-larvae | 30 | 2.02 | 1.2e-03 |
| efl-1 | EFL-1\_Larvae-L1-stage | 26 | 2.14 | 2.2e-03 |
| nhr-77 | NHR-77\_Larvae-L4-stage | 33 | 1.84 | 2.4e-03 |
| dve-1 | DVE-1\_Late-Embryos | 22 | 2.31 | 3.5e-03 |
| F16B12.6 | F16B12.6\_Fed-L1-stage-larvae | 15 | 3.01 | 3.9e-03 |
| R02D3.7 | R02D3.7\_Larvae-L3-stage | 26 | 2.05 | 4.2e-03 |
| lin-35 | LIN-35\_Fed-L1-stage-larvae | 23 | 2.21 | 4.2e-03 |
| gei-11 | GEI-11\_Fed-L1-stage-larvae | 26 | 2.05 | 4.3e-03 |
| eor-1 | EOR-1\_Larvae-L3-stage | 26 | 2.05 | 4.4e-03 |
| nhr-23 | NHR-23\_Larvae-L3-stage | 20 | 2.42 | 4.4e-03 |
| ham-1 | HAM-1\_Fed-L1-stage-larvae | 25 | 2.09 | 4.6e-03 |
| ham-1 | HAM-1\_Larvae-L4-stage | 26 | 2.03 | 5.2e-03 |
| nfya-1 | NFYA-1\_Late-Embryos | 25 | 2.07 | 5.3e-03 |
| dpl-1 | DPL-1\_Larvae-L4-stage | 30 | 1.86 | 5.4e-03 |
| lsy-2 | LSY-2\_Fed-L1-stage-larvae | 24 | 2.12 | 5.4e-03 |
| efl-1 | EFL-1\_Fed-L1-stage-larvae | 23 | 2.17 | 5.5e-03 |
| pes-1 | PES-1\_Larvae-L4-stage | 28 | 1.93 | 5.8e-03 |
| ztf-7 | ZTF-7\_Larvae-L4-stage | 15 | 2.82 | 7.4e-03 |
| F45C12.2 | F45C12.2\_Fed-L1-stage-larvae | 22 | 2.17 | 7.9e-03 |
| C01B12.2 | C01B12.2\_Larvae-L2-stage | 30 | 1.82 | 8.3e-03 |
| lsy-2 | LSY-2\_Larvae-L1-stage | 30 | 1.81 | 8.9e-03 |
| nhr-6 | NHR-6\_Larvae-L4-stage | 16 | 2.50 | 1.6e-02 |
| R02D3.7 | R02D3.7\_Larvae-L2-stage | 15 | 2.58 | 1.7e-02 |
| fos-1 | FOS-1\_Fed-L1-stage-larvae | 22 | 2.01 | 2.2e-02 |
| lin-13 | LIN-13\_Larvae-L2-stage | 18 | 2.23 | 2.4e-02 |
| lin-15 | LIN-15B\_Fed-L1-stage-larvae | 15 | 2.47 | 2.7e-02 |
| nhr-129 | NHR-129\_Larvae-L2-stage | 24 | 1.83 | 3.9e-02 |
| efl-1 | EFL-1\_Young-adult | 26 | 1.76 | 4.0e-02 |
| jun-1 | JUN-1\_Larvae-L4-stage | 16 | 2.24 | 4.5e-02 |
